# Investigation of syphilis immunology and Treponema pallidum subsp. pallidum biology to improve clinical management and design a broadly protective vaccine: study protocol

**DOI:** 10.1186/s12879-020-05141-0

**Published:** 2020-06-23

**Authors:** Ethan Osias, Phoebe Hung, Lorenzo Giacani, Chrysovalantis Stafylis, Kelika A. Konda, Silver K. Vargas, E. Michael Reyes-Díaz, W. Scott Comulada, David A. Haake, Austin M. Haynes, Carlos F. Caceres, Jeffrey D. Klausner

**Affiliations:** 1 Division of Infectious Diseases, David Geffen School of Medicine, University of California Los Angeles, 10833 Le Conte Ave, Los Angeles, CA 90095 USA; 2 Department of Medicine, Division of Allergy and Infectious Diseases, and Department of Global Health, University of Washington, Seattle, WA USA; 3 Unit of Health, Sexuality and Human Development and Laboratory of Sexual Health, Universidad Peruana Cayetano Heredia, Lima, Peru; 4 Veterans Affairs Greater Los Angeles Healthcare System, Los Angeles, CA USA

**Keywords:** Syphilis, *Treponema pallidum*, Peru, Cytokine profiling, Molecular typing, Vaccine development

## Abstract

**Background:**

The syphilis epidemic continues to cause substantial morbidity and mortality worldwide, particularly in low- and middle-income countries, despite several recent disease control initiatives. Though our understanding of the pathogenesis of this disease and the biology of the syphilis agent, *Treponema pallidum* subsp. *pallidum* has improved over the last two decades, further research is necessary to improve clinical diagnosis and disease management protocols. Additionally, such research efforts could contribute to the identification of possible targets for the development of an effective vaccine to stem syphilis spread.

**Methods:**

This study will recruit two cohorts of participants with active syphilis infection, one with de novo infection, one with repeat infection. Whole blood specimens will be collected from each study participant at baseline, 4, 12, 24, 36, and 48 weeks, to track specific markers of their immunological response, as well as to compare humoral reactivity to *Treponema pallidum* antigens between the two groups. Additionally, we will use serum specimens to look for unique cytokine patterns in participants with early syphilis. Oral and blood samples, as well as samples from any syphilitic lesions present, will also be collected to sequence any *Treponema pallidum* DNA found.

**Discussion:**

By furthering our understanding of syphilis pathogenesis and human host immune response to *Treponema pallidum*, we will provide important data that will help in development of new point-of-care tests that could better identify active infection, leading to improved syphilis diagnosis and management. Findings could also contribute to vaccine development efforts.

## Background

Syphilis is an important public health issue worldwide. The World Health Organization has estimated that there are 5.6 million new cases of syphilis globally each year [1], and global prevalence is estimated to be 56.1 million [2]. In the United States alone, syphilis incidence has more than tripled since 2000 [3]. Congenital syphilis rates in the US have also increased and are currently the highest since 1997 [3]. Globally, syphilis impacts more pregnancies than the human immunodeficiency virus (HIV), with an estimated 351,000 adverse pregnancy outcomes due to syphilis every year, including 205,000 early fetal and neonatal deaths, and 146,000 babies born preterm, with low birthweight, or with signs of infection [4]. Syphilis infection also increases the risk of HIV acquisition and transmission [5]. This is especially prevalent in key populations such as men who have sex with men (MSM), as 45.5% of MSM with syphilis in the United States are co-infected with HIV [3].

Currently, the diagnosis and treatment of syphilis rely on immunological markers and clinical management protocols that have not been substantially improved in more than 75 years [6–10]. Most syphilis testing protocols rely on the combination of a treponemal (presence of antibodies to *Treponema pallidum* antigens) and a non-treponemal test (presence of antibodies against lipoidal antigens). This is an imperfect process with a reliance on historic information such as previous rapid plasma reagin (non-treponemal) or treponemal antibody test results built into the algorithm. Treatment, which consists of varying regimens of intramuscular benzathine penicillin [8], while effective, increases in time and discomfort in the absence of documentation of previous test results. As a result, well-trained doctors often have difficulty diagnosing and treating syphilis due to deficiencies in the historic information available to them. In addition to the clinical disadvantages, the current testing protocols also increase public health resource-draining activities such as case finding.

The biology of the syphilis-causative bacterium *Treponema pallidum* has been further elucidated in the last decades, setting the stage for advancements in rapid test development that could address the current shortcomings in clinical management. The availability of pathogen genomes has allowed for the identification of several bona fide and putative *Treponema pallidum* surface-exposed outer membrane proteins that likely play a key role in the host-pathogen interplay during infection, as they are known immunogens [11–13]. Through comprehensive cytokine analysis and characterization of serum antibody responses to specific *Treponema pallidum* antigens, our research looks to determine if there is a difference in syphilis pathogenesis and human immune response in participants with de novo versus repeat infection. We also look to elucidate specific immune markers that can be used in the development of new diagnostic tests. This would improve syphilis clinical management and also possibly resolve the clinical question of whether patients who maintain a persistently low rapid plasma reagin titer (known as serofast patients) need treatment [14–17].

Syphilis incidence in countries of every income level continues to rise, especially among MSM [18], despite the implementation of successful screening and prevention programs in the United States and globally, warranting new research efforts specifically working towards development of a vaccine [19]. A safe and effective syphilis vaccine could drastically reduce the global burden of syphilis disease and potentially lead to syphilis elimination worldwide [19]. Our study looks to contribute to vaccine development efforts through the further identification and characterization of *Treponema pallidum* antigens that play a role in syphilis pathogenesis, building on prior work done by other researchers [20].

In 2012, as part of a National Institutes of Health research capacity development program [21], our group initiated a longitudinal cohort study of syphilis in Peru, called the PICASSO study, of over 400 MSM and transgender women [22]. This study successfully enrolled, followed quarterly, and retained 77% of a cohort of 401 high-risk men who have sex with men and transgender women over 24 months [23, 24]. We strengthened our sexual health laboratory with the capacity to conduct molecular-based *Treponema pallidum* DNA studies [25–27], and created a biospecimen repository of over 3000 serum and clinical specimens. We also conducted evaluations of multiple new commercial point-of-care rapid immunoassays [28–32], created clinical research infrastructure at two sexual health clinics in Lima to recruit, screen, enroll and reliably assess and retain participants, and conducted epidemiologic and clinical/immunological evaluations including positron emission tomography scanning [33, 34] and novel serum cytokine analyses [35, 36].

Our current study builds on our previous work, bringing together experts in *Treponema pallidum* genomics, proteomics, and syphilis pathogenesis to understand and predict the molecular mechanisms of spirochete pathogenesis and immunity mediated by outer membrane proteins [37]. This expertise will be utilized to address inadequacies in syphilis diagnosis and treatment by studying the immunological differences in syphilis pathogenesis in participants with de novo versus repeat infection. Here we describe a study that will take place over 4 years in Lima, Peru. We will recruit and treat two cohorts of participants with recently acquired syphilis infection, one with de novo infection and one with repeat infection. We hypothesize that there are immune markers in syphilis pathogenesis that are unique to individuals in each of the two cohorts and that those markers may prove useful for the development of new rapid tests and potentially contribute knowledge towards the development of a vaccine.

## Methods/design

### Overview

We look to study a population with current, active syphilis infection in Lima, Peru. Based on the previous PICASSO study, the prevalence of recently acquired syphilis in this population is expected to be as high as 16.8% in men who have sex with men and 6.7% in transgender women [38]. Comparatively, baseline prevalence in the United States is generally much lower with an incidence of 9.5 cases per 100,000 population [3]. Consequently, studying syphilis infection in this setting provides ample opportunity to explore the clinical epidemiology of syphilis, the immunologic response to the pathogen, and the molecular profile of disease pathogenesis.

The primary objectives of this study are two-fold. First, in terms of clinical epidemiology, we will recruit, treat, and follow individuals with incident syphilis infection both with and without prior syphilis infection (i.e. de novo infection and repeat syphilis infection). Comorbid HIV infection is common in syphilis patients [3], so we will also identify and refer for treatment HIV-infected individuals and group them separately. We will follow these groups over time and compare their markers of immunologic response. Secondly, we will investigate whether a relationship exists during early syphilis between differential gene expression in *Treponema pallidum*, development of the immune response to treponemal antigens, host cytokine profiles of disease pathogenesis and immune correlates of infection. We hypothesize that the clinical and immunologic response will differ between individuals with repeat syphilis infection versus de novo incident infection as well as between active versus treated infection. We also predict the response will differ based on HIV-associated immunosuppression and *Treponema pallidum* strain.

To fulfill these aims, we will recruit and treat 200 participants with recently acquired syphilis infection, 100 with de novo infection and 100 with repeat syphilis infection. These participants will be assessed at baseline, 4, 12, 24, 36, and 48 weeks (Additional file 1). Samples will be taken at each visit for molecular and cytokine measurement for the purposes stated above. The study protocol has been approved by the institutional review boards at Universidad Peruana Cayetano Heredia and University of California, Los Angeles and covers all five study sites in Lima, Peru.

### Study sites

Participants from the study will be recruited from five sexual health clinics across Lima, Peru – Centro de Salud Alberto Barton (Barton), Centro Materno Infantil Tahuantinsuyo bajo (Tahuantinsuyo), CERITS Tres Compuertas de Caja de Agua (Caja de Agua), Epicentro, UAMP del Hospital Amazónico (Pucallpa). These centers serve as sexual health clinics that are well known among the general population and easily accessible to local residents, including men who have sex with men, transgender women, and sex workers. They were chosen due to a high incidence of syphilis infection, as well as the capacity of their laboratories to accommodate the needs of the study [22]. It was imperative that each site be able to run rapid treponemal tests, but more importantly provide on-site and real time rapid plasma reagin testing.

The participants will be recruited from the sexual health clinics over 18 months. We are still in the recruitment stage and expect to see 1000 patients with active syphilis in order to enroll our 200-participant cohort, based on the syphilis incidence at each site among individuals with past clinical information.

### Study groups

The study will recruit two cohorts of individuals: 1) 100 participants with new (de novo) syphilis infection and 2) 100 participants with repeat syphilis infection. A new syphilis infection is defined as a newly reactive rapid plasma reagin, a *Treponema pallidum*-particle agglutination confirmed positive, and a documented *Treponema pallidum* negative antibody test taken within the past 12 months at the sexual health facility. Existing health records must include the syphilis testing history. A repeat syphilis infection is defined by a new four-fold rise or newly reactive rapid plasma reagin when previously rapid plasma reagin was seronegative, with a documented *Treponema pallidum* antibody positive test at the sexual health facility.

### Eligibility criteria

Participants who are 18 years or older and are newly diagnosed with syphilis will be invited to join the study if they meet the criteria outlined in Table 1.

**Table 1:** Inclusion and exclusion criteria for the PICASSO 2 study, Lima, Peru, 2018–2022

Inclusion Criteria	Exclusion Criteria
Age ≥ 18 years old	Unable to give informed consent
Newly diagnosed with syphilis infection	Unlikely to complete study follow up
• Group 1 (*n* = 100): No history of syphilis and documented negative Treponema pallidum antibody test in past 12 months	
• Group 2 (*n* = 100): History of syphilis (documented positive Treponema pallidum antibody test) and history of syphilis treatment	
Intention to stay in Lima or its environs for the next 12 months	
Willingness to provide fingerstick and venipuncture whole blood specimens	

### Screening and enrollment

**Fig. 1:**
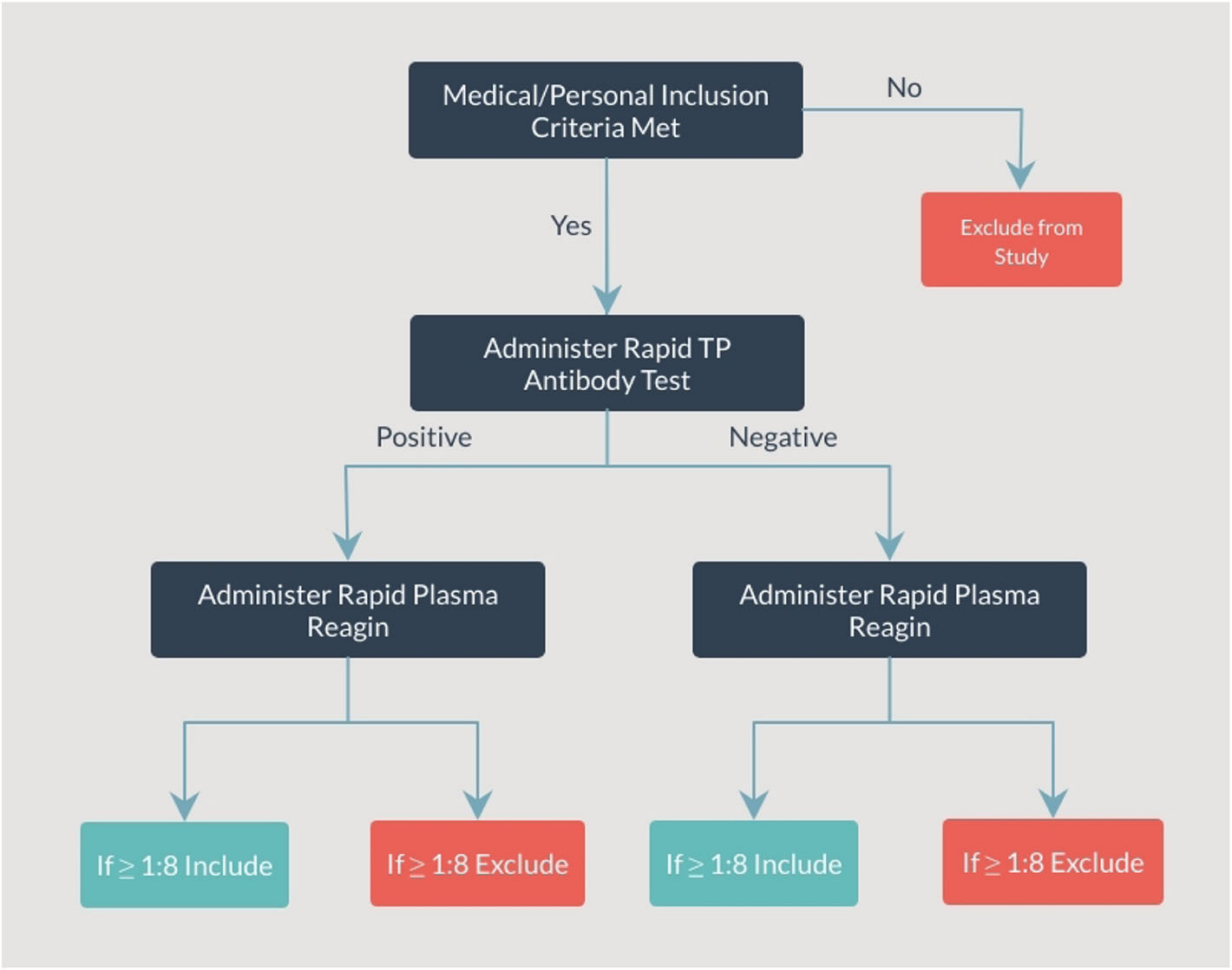
Enrollment algorithm for PICASSO 2 study, Lima, Peru, 2018–2022

At each study clinic, the site coordinator will approach patients recently diagnosed with syphilis. The site coordinator will inform them of the study, review all study procedures, and request permission to screen them for eligibility using the inclusion/exclusion criteria. Interested participants will be informed about the risks, benefits and alternatives of joining the study. Participants will be enrolled based on the algorithm found in Fig. 1. The research assistant will obtain signed informed consent from all participants and clarify that participants can opt out from participating in the study without change to their clinical care. Contact information for the investigator and local institutional review board will also be provided.

All syphilis infections will be treated on-site per CDC STI Treatment Guidelines [8]. The results of HIV and syphilis tests will be given to participants using appropriate post-test counseling. Participants with neurologic symptoms will be referred to a separate diagnostic and treatment center.

After enrollment, participants will be followed and retained using weekly text messages, follow-up calls, and notifications. These methods were previously successful in obtaining an overall retention rate of 84% at 2 years post-baseline assessment in our target population [39].

### Specimen collection

Because the aim of this study is to document the human host immune response to *Treponema pallidum*, we will be utilizing several tests to characterize the immunological response over time in the de novo infection group and the repeat infection group. The type and frequency of tests administered to each patient at follow up are detailed in Table 2.

**Table 2:** Follow-up testing and specimen collection for all participants in the PICASSO 2 study by time of visit, Lima, Peru, 2018–2022

Test	Baseline	4 weeks	12 weeks	24 weeks	36 weeks	48 weeks
**Rapid*Treponema pallidum*antibody test**	x					
**Rapid plasma reagin**^**a**^	x	x	x	x	x	x
***Treponema pallidum*-particle agglutination test**^**b**^	x	x	x	x	x	x
**Whole blood and serum collection**	x	x	x	x	x	x
**Rapid HIV test**	x	x (if previously HIV uninfected)	x (if previously HIV uninfected)	x (if previously HIV uninfected)	x (if previously HIV uninfected)	x (if previously HIV uninfected)
**Absolute CD4 count (if HIV infected)**	x	x	x	x	x	x
**Plasma viral load (if HIV infected)**	x	x	x	x	x	x
**Lesion swab**	x					
**Oropharyngeal swab**	x					

^a^The cut-off value for current infection will be set at ≥1:8

^b^The *Treponema pallidum*-particle agglutination test will be used to confirm rapid plasma reagin results using a reactive cutoff value of ≥1:80

For rapid treponemal testing, the Abbott Determine™ Rapid Syphilis TP assay (Determine™ Syphilis TP, Abbott, US) will be used to determine lifetime history of syphilis infection. For non-treponemal testing, rapid plasma reagin (RPR Quicktest, Stanbio™, US) was chosen instead of the traditional Venereal Disease Research Laboratory test because it provides a slightly higher sensitivity and specificity without the need of a microscope [40]. Rapid plasma reagin will be used to establish current infection and will be quantified to obtain a titer. Individuals with a titer of 1:8 or greater will be included, even if their rapid TP test is negative, and those with a titer under 1:8 will be excluded. The rapid plasma reagin result will be confirmed with a *Treponema pallidum*-particle agglutination test (SERODIA®-TP-PA, Fujirebio, US) at the Universidad Peruana Cayetano Heredia Sexual Health Lab using a reactive cutoff value of ≥1:80.

We will also collect lesion and oropharyngeal swab samples for *Treponema pallidum* DNA and RNA isolation and strain typing, and serum specimens for cytokine determination. Oropharyngeal swabs have been demonstrated to contain *Treponema pallidum* DNA in early syphilis patients and can be used as a source for treponemal DNA testing [41], thus providing more comprehensive *Treponema pallidum* DNA detection and strain characterization [42].

All participants will also be administered a rapid HIV test (Determine™ HIV-1/2 Ag/Ab Combo, Abbott, US, or HIV 1/2 Ab Plus Combo Rapid Test, CTK Biotech, US); and their absolute CD4 T-cell counts will be determined (Alere Pima™ Analyser, Abbott, US). Plasma viral load will also be determined in participants with positive rapid HIV test result (Xpert® HIV-1 Viral Load, Cepheid, US).

Clinical results will be given to HIV-infected participants 7–10 days after the initial visit, this includes the HIV confirmatory test performed at the initial visit, CD4 T cell count, and viral load results. Individuals diagnosed with HIV infection will be referred to appropriate services as per clinic protocol and the Peruvian National HIV Treatment Program. Research data unrelated to clinical care will not be returned to participants.

In addition, we aim to continue our work in describing unique cytokine patterns in participants with early syphilis. To this end, we will use serum samples from participants at baseline and at each follow-up visit after the date of syphilis diagnosis. Whole blood will be drawn into silicon-coated Vacutainer tubes, separated by centrifugation, and serum frozen at − 80 °C. Specimens will be kept frozen until used to test for expression of cytokines using a 62-plex cytokine assay system. The cytokine panel will include known Th1/Th2 cytokines (such as IL-2, IL-4, IL-6, IL-8, IL-10, IL-17, IFN-gamma, and TNF-alpha (Luminex multiplex assay, Millipore, US)). All measurements will be performed in duplicate, masked from clinical data. Values < 2 pg/mL will be assigned 2 pg/mL. Molecular typing of the *Treponema pallidum* strains present in blood, or lesion/tissue swabs will be performed on samples that initially screen positive for the presence of *tp0574* DNA (the gene encoding the 47 KDa lipoprotein of *Treponema pallidum*). Typing will be performed according to the enhanced CDC protocol as we have previously described [27].

### Molecular testing

Significant work has been done both before and after the genome sequence of *Treponema pallidum* became available to characterize *Treponema pallidum* proteins both functionally and immunologically, with particular attention to lipoproteins, putative surface-exposed proteins, and ligand binding proteins. Lipoproteins are known to be potent immune-stimulatory antigens during syphilis infection [43–47] due to the ability of their acyl-moiety to stimulate Toll-like receptor 2 receptors, while ligand binding proteins likely play a central role in *Treponema pallidum* physiology and biology, given the extreme dependence of the syphilis agent on the host for nutrient acquisition [48]. Surface-exposed proteins are the most likely targets for protective immunity during syphilis infection [49–51]. Although unanimous consensus in the field about the identity of *Treponema pallidum* surface-exposed proteins is still missing, due to the difficulty of direct identification of these proteins on *Treponema pallidum* surface, sufficient indirect evidence has been accumulated on a subset of antigens to support their surface exposure [13, 52–56]. Most importantly, all the selected lipoproteins, ligand binding proteins, and putative surface-exposed proteins are already known to elicit an immune response during experimental and natural syphilis infection [11, 15].

The Giancani Laboratory, led by one of our team members, has been involved for almost two decades in expressing *Treponema pallidum* recombinant antigens and studying the immune response to several of the lipoproteins and surface-exposed proteins [13, 49, 52, 57] that will be used in our study. For this study we will use an enzyme-linked immunosorbent assay purposely developed for this study to investigate the humoral response to a panel array of 15 *Treponema pallidum* lipoproteins, surface-exposed proteins and ligand binding proteins, selected based on their immunogenicity, and potential for differential expression in different stages of early infection (primary, secondary and early latent). Those *Treponema pallidum* antigens have been summarized in Table 3.

**Table 3:** *Treponema pallidum* antigens (15-plex immune array) for immunological studies in Lima, Peru, 2018–2022

Lipoproteins	Putative surface-exposed proteins	Ligand binding proteins
TP0435 (Tpp17)	TP0126 (OmpW homolog)	TP0163 TroA (Periplasmic binding protein)
TP0574 (47 kDa Carboxypeptidase)	TP0136 (FN-binding protein)	TP0684 MglB-2 (Periplasmic binding protein)
TP0751 (Pallilysin)	TP0326 (BamA)	TP1038 (Bacterioferrin)
TP0768 (TmpA)	TP0483 (FN-binding protein)	
TP0769 (TmpB)	TP0620 (TprI)	
	TP0621 (TprJ)	
	TP0897 (TprK conserved region)	

We will use those antigens, to investigate and possibly elucidate changes in the humoral response to *Treponema pallidum* in participants with new or repeat infection. To this end, antigens will be expressed using recombinant DNA technology in *Escherichia coli* purified by nickel-affinity and size-exclusion chromatography. Proteins that cannot be expressed as soluble antigens will be purified under denaturing conditions using chaotropic agents such as urea orguanidine HCl and dialyzed against a buffer containing proteins stabilizers (e.g. arginine HCl) and detergents (e.g. OTG, BriJ-35, or glycerol) to avoid protein precipitation. Antigens will be organized on enzyme-linked immunosorbent assay plates and used for antibody detection in enzyme-linked immunosorbent assay format as already described [13]. Levels of target-specific antibodies will be compared for the same stage as well as between different stages of syphilis, and in new versus repeat infections.

Based on previous work by other investigators [11, 15], in new syphilis cases we expect to see reactivity against TP0435 to be similar in primary and secondary syphilis and decline in early latent cases. Reactivity to TP0574, TP0769, and TP0136 is expected to be highest during primary syphilis and quickly decline in subsequent stages, while reactivity against TP0768 and TP0684 should be similar in primary and early latent syphilis but lower in secondary syphilis sera. Reactivity against TP0163 and TP1038 should be highest in early latent cases, compared to primary or secondary cases. Response to the other antigens should fall within one of the above groups. We will also determine if the humoral responses detected here correlate with the messenger RNA level for most *Treponema pallidum* genes that we will describe later on in this study.

Furthermore, we will investigate whether a relationship exists during early syphilis between differential gene expression in *Treponema pallidum*, development of the immune response to treponemal antigens, and host cytokine profiles to identify molecular markers of disease pathogenesis and novel vaccine candidates. Identification of these markers could provide important information that paves the way for major advancements in syphilis point-of-care testing, leading to more accurate diagnosis, better informed treatment, public health resource conservation, and prevention.

The molecular markers will be identified through samples obtained from swabs of syphilis lesions on the mucosal tissue or skin from enrolled participants with primary or secondary lesions. Collected samples will be stored in a -80 °C freezer, and shipped to the Giacani laboratory at the University of Washington. Total RNA will be extracted from the samples using the *Quick*-RNA Kit (Zimo Research, US), which includes a DNase incubation step to eliminate residual genomic DNA. This procedure will be followed by mRNA enrichment using the MicrobExpress and MicrobEnrich kits (Life Technologies), reverse transcription, total RNA amplification using a SeqPlex RNA Amplification Kit (Sigma) and library preparation.

Treponemal lipoproteins are known to be the main antigens recognized by host Toll-like receptors during syphilis infection [58]. Because of this, we anticipate that comparative analysis of *Treponema pallidum* transcriptomes will reveal differences in transcription of virulence-associated genes encoding for *Treponema pallidum* lipoproteins and potentially other antigens as well. In particular, we expect that LP expression will positively correlate with the level of Toll-like receptor 2-mediated proinflammatory cytokines such as TNF-α, IL-1β, IL-6, and IL-12.

We also expect to see transcriptional modulation of genes controlled by *Treponema pallidum* only extra-cytoplasmic function sigma factor, TP0092. Extra-cytoplasmic sigma factor is a transcription factor capable of controlling large groups of genes and is involved in counteracting host-induced environmental stresses and likely fostering *Treponema pallidum* adaptation to different anatomical micro-environments, contributing to persistence in the host [59–67]. We also expect transcriptional modulation of several of the pathogen’s motility and chemotaxis genes, and genes that are involved in detoxification of reactive oxygen species also shown by Giacani et al. to be controlled by an extra-cytoplasmic sigma factor [68].

It is known that during primary experimental syphilis, treponemes are cleared from the primary lesion by opsono-phagocytosis following the appearance of anti-*Treponema pallidum* specific antibodies [69]. Despite this, a sub-population of treponemes resistant to macrophage ingestion emerges and persists [70], suggesting that treponemes modify the availability of surface proteins targeted by opsonic antibodies. Based on this evidence, we expect to find a selective decrease in transcription of several, but not all, genes encoding *Treponema pallidum* putative outer membrane proteins in samples harvested from participants with high titers of treponemal antibodies. Such differential expression could be a contributing mechanism to the increased resistance to opsono-phagocytosis of the syphilis agent. As mentioned, however, we do not expect to see down-regulation of all *Treponema pallidum* putative outer membrane proteins.

Giacani et al. also reported that transcription of TprK, a putative *Treponema pallidum* outer membrane protein, does not change throughout the course of primary experimental syphilis [49]. This is likely possible because the TprK protein escapes immune detection via antigenic variation of its surface exposed regions [58, 71, 72], and its expression does not necessarily need to be downregulated during infection. We also expect to see variability in expression of additional putative outer membrane proteins with proteolytic activity, and ability to bind the host extracellular matrix components such as Tp0751 or Tp0136, which are involved in adhesion to host tissues, invasion, and dissemination [73, 74].

### Statistical analyses

Comprehensive descriptive and exploratory analyses will be conducted with information collected before testing our hypotheses of clinical and immunologic response differences between individuals with repeat infection versus de novo syphilis infection.

Primary analyses will use random-effects regression models to evaluate the impact of de novo versus repeat infection on immune markers of syphilis pathogenesis, including epitope immunoassay responses, rapid plasma reagin patterns, and cytokine profiles. Appropriate regressions will be used for the type of outcome modeled. For example, rapid plasma reagin titers will be classified as non-reactive, 1:1, 1:2, and so forth in an ordered fashion; random-effects ordinal regression will be used. Models will include random effects to model repeated outcome measurements on the same study participants.

Given the likely frequent presence of HIV co-infection (20–40%) [75], HIV-infection will be included as a key three-category confounder in all analyses: 1) individuals without HIV-infection, 2) those with controlled HIV-infection (i.e. with undetectable viral load), and 3) those with uncontrolled HIV-infection (i.e. those with detectable viral load > 200 copies/ml). All analyses will also include covariates to account for linear or nonlinear trends in outcomes over time, based on formal statistical tests and visual inspection of plots that show outcome trajectories over time. Clinic-level fixed effects will also be included to account for potential outcome heterogeneity across health clinics. Since multiple outcomes will be evaluated, we will use methodology outlined in Harwood et al. [76] to estimate an overall effect of de novo versus repeat syphilis infection on immune marker responses. The methodology preserves type I error by estimating the number of statistically significant effects that would be expected from individual regression models under the null hypothesis in the presence of correlated outcomes.

### Sample size and power calculations

Sample size calculations were carried out using RMASS [77] to compare outcomes over time between participants with de novo (*n* = 100) versus repeat syphilis infection (*n* = 100). We estimate that a sample size of 200 participants will yield reasonable effect sizes across outcomes. For example, outcomes that will be analyzed as continuous variables, such as mRNA levels, will yield an effect size of 0.63 at the last time point over 48 weeks. Calculations assume 80% power at a 0.05 two-sided significance level and an autocorrelation of 0.5 between repeated outcome measurements.

## Discussion

This study looks to compare immune markers of syphilis pathogenesis in individuals with de novo versus repeat syphilis infection and use the findings towards identification of novel antigens that might be used in rapid tests as well as differentiate between active and repeat infection. Additionally, a better understanding of syphilis pathogenesis may help contribute to development of a vaccine.

We expect the benefits of this study to outweigh the risks. Physical risks from procedures such as blood collection will be minimized by utilizing trained phlebotomists and universal precautions. The primary risks to participants are psychological and social. We will minimize psychological damage that can result from the diagnosis of stigmatizing diseases by educating participants about the nature and consequences of syphilis and/or HIV infection and treatment, and by providing treatment or referral for treatment for those who test positive as per standard treatment protocols. We will minimize social risks such as potential release of sensitive information by using study identification numbers instead of names, and by storing study records in locked filing cabinets, password protecting devices, and encrypting files. Additionally, study personnel and clinic personnel working where study activities will occur are properly trained to keep study participation private, and are well versed in post-test counseling for STIs.

Overall, an improved understanding of the pathogenesis of syphilis will be beneficial because it might help develop novel diagnostic approaches that could improve care for syphilis patients. The data on the immunological response to *Treponema pallidum* antigens may also provide information on possible vaccine targets. The development of such a vaccine could bring us significantly closer to syphilis disease control.

## Supplementary information

**Additional file 1.**

## Data Availability

The datasets from the current study are not publicly available as the study is ongoing and data are still being collected. Data collected thus far will be made available from the corresponding author on reasonable request.

## Abbreviations

CDC: Center for Disease Control
DNA: Deoxyribonucleic acid;
HIV: Human immunodeficiency virus
RNA: Ribonucleic acid
STI: Sexually transmitted infection
