# Supplementary material for: Investigation of syphilis immunology and Treponema pallidum subsp. pallidum biology to improve clinical management and design a broadly protective vaccine: study protocol
Source: BMC Infect Dis. 2020 Jun 23;20:444. doi: 10.1186/s12879-020-05141-0 (PMC7309211; doi:10.1186/s12879-020-05141-0)
Supplement: Supplementary file 1 — Additional file 1. [file 12879_2020_5141_MOESM1_ESM.docx]

**CLINICAL FORM - BASAL, version 5**

1. **General information**

| Evaluator initials: ___________  (Write initials of first name and 2 last names) | Paste ID label here |
| --- | --- |
| Location :  Ο Barton Ο Tahuantinsuyo Ο Caja de agua Ο Epicentro Ο Pucallpa | |
| Visit date : __ __ __ __ / __ __ / __ __  *Y Y Y Y M M D D* | |

1. **Morbidities**

| 1. Does the participant have any acute or chronic illness?   (Including previously diagnosed HIV) | Ο Yes Ο No  ***(If “No” jump to P 2)*** |  |
| --- | --- | --- |
| - 1. Write the illnesses the participant has: |  |  |
| ________________________________________________________________________________________  ________________________________________________________________________________________  ________________________________________________________________________________________ | |  |

1. **HIV infection history**

| 1. Is the participant HIV positive? | Ο Yes Ο No  ***(If “No” jump to P 3)*** |
| --- | --- |
| - 1. Is the participant´s first HIV result registered in the clinical record? | Ο Yes Ο No  ***(If “No” jump to P 2.2)*** |
| - - 1. Date of first HIV positive result in medical record | Date: __ __ __ __ / __ __ / __ __  *Y Y Y Y M M D D* |
| - 1. Is the participant’s first CD4 count result registered in the clinical record? | Ο Yes Ο No  ***(If “No” jump to P 2.2)*** |
| - - 1. Which is the result and date of the participant’s first CD4 count? | |
| CD4 count: _________________________  (Numeric values only) | Date: __ __ __ __ / __ __ / __ __  *Y Y Y Y M M D D* |
| - 1. Is the participant’s first viral load test result registered in the clinical record? | Ο Yes Ο No  ***(If “No” jump to P 2.3)*** |
| - - 1. Which is the result and date of the participant’s first viral load test? | |
| Viral Load: _________________________ | Date: __ __ __ __ / __ __ / __ __  *Y Y Y Y M M D D* |
| - 1. Is the participant currently on HIV treatment?   *(Take information from medical record. If there is no information on medical record, ask the participant)* | Ο Yes Ο No  ***(If “No” jump to P 3)*** |

| - - 1. Current antirretroviral regimen:   *(Take information from medical record. If there is no information on medical record, ask the participant)*   - ATRIPLA: Tenofovir 300 mg (TDF)/Emtricitabina 200 mg (FTC)/Efavirenz 600 mg (EFV)**, jump to P 3** - Tenofovir 300 mg (TDF)+Lamivudina 150 mg (3TC) +Efavirenz 600 mg (EFV)**, jump to P 3** - TRUVADA +EFAVIRENZ: Tenofovir 300 mg (TDF)/Emtricitabina 200 mg (FTC) +Efavirenz 600 mg (EFV)**, jump to P 3** - Another**, complete regimen in question P 2.4.1.1** - No information on medical record/Participant doesn’t remember**, jump to P 3** |
| --- |
| - - - 1. Specify other antiretroviral regimen: ________________________________________   ________________________________________________________________________________________________________________________________________________________________  *(Complete drug´s names and doses from clinical record. If there is no information on clinical record, ask the participant)* |

1. **Syphilis history**

| 1. Did the participant have syphilis in the past? | | | | Ο Yes Ο No Ο Don’t know  (If “No” or “Don’t know” jump to **P 4)** | | |
| --- | --- | --- | --- | --- | --- | --- |
| - 1. Complete RPR test result (dilutions) from last syphilis diagnosis | | | | | | |
| Ο No reactive | Ο 1:1 | Ο 1:2 | Ο 1:4 | Ο 1:8 | Ο 1:16 | Ο 1:32 |
| Ο 1:64 | Ο 1:128 | Ο 1:256 | Ο 1:512 | Ο 1:1024 | Ο No information on clinical record**, jump to P 3.2** | |
| - - 1. Complete date of RPR test result from last syphilis diagnosis | | | | Date: __ __ __ __ / __ __ / __ __  *Y Y Y Y M M D D* | | |
| - 1. Complete last TP rapid test result | | | | Ο Reactive  Ο No reactive  Ο No information on clinical record**, jump to P 3.3** | | |
| - - 1. Complete date of last TP rapid test result | | | | Date: __ __ __ __ / __ __ / __ __  *Y Y Y Y M M D D* | | |
| - 1. Complete last registered RPR test result (dilutions)   If there isn’t a RPR test after last syphilis diagnosis, complete with RPR result and date from last syphilis diagnosis | | | | | | |
| Ο No reactive | Ο 1:1 | Ο 1:2 | Ο 1:4 | Ο 1:8 | Ο 1:16 | Ο 1:32 |
| Ο 1:64 | Ο 1:128 | Ο 1:256 | Ο 1:512 | Ο 1:1024 | Ο No information on clinical record, **jump to *P 3.*** | |
| - - 1. Complete date of last RPR test result | | | | Date: __ __ __ __ / __ __ / __ __  *Y Y Y Y M M D D* | | |
| - 1. How many times have the participant been diagnosed with Syphilis? | | | | ___________  (Numeric values only) | | |
| - 1. How many times have the participant been treated for Syphilis? | | | | ___________  (Numeric values only) | | |
| - 1. Which was the last syphilis treatment regimen the participant received?   *(Take information from medical record. If there is no information on medical record, ask the participant)* | | | | | | |
| Ο Penicillin G Benzathine 1 dose, jump to **P 3.6.2 (Date from last treatment)**  Ο Penicillin G Benzathine 3 doses, jump to **P 3.6.2 (Date from last treatment)**  Ο Doxycycline 100mg 2 weeks, jump to **P 3.6.2 (Date from last treatment)**  Ο Doxycycline 100mg 4 weeks, jump to **P 3.6.2 (Date from last treatment)**  Ο Another treatment, **complete another treatment in question P 3.6.1**  Ο Do not remember/No in clinical record, jump to **P 3.6.2 (Date from last treatment)**  Ο Never treated, **jump to P 3.7** | | | | | | |
| - - 1. Which another treatment did the participant receive?   __________________________________________________________________  __________________________________________________________________  __________________________________________________________________  *(Complete drug´s names and doses from clinical record. If there is no information on clinical record, ask the participant)* | | | | | | |
| - - 1. Last syphilis treatment date | | | | Date: __ __ __ __ / __ __ / __ __  *Y Y Y Y M M D D* | | |
| - 1. Within 24 hours after taken/receiving syphilis treatment, did the participant had new or more intensive symptoms of fever, malaise or cutaneous rash? | | | | Ο Yes Ο No | | |
| 1. Is the participant allergic to Penicillin? | | | | Ο Yes Ο No Ο Don’t know | | |
| 1. Does the participant have any autoimmune disease such as lupus or rheumatoid arthritis? | | | | Ο Yes Ο No | | |

1. **Primary syphilis symptoms**

|  | **Yes** | **No** | **Doesn’t know** |
| --- | --- | --- | --- |
| 1. Has there been any ulcer in the oral region?   ***(In the last 2 weeks)*** |  | *Jump to P7* | *Jump to P7* |
| - 1. Was the ulcer painful? |  |  |  |
| - 1. Did you receive treatment for the ulcer? |  | *Jump to P7* | *Jump to P7* |
| - - 1. Specify treatment:   ____________________________________________________________________________________ | | | |
| - - 1. Specify treatment date. __ __ __ __ / __ __ / __ __   *Y Y Y Y M M D D* | | | |
| 1. Has there been any ulcer in the genital region?   ***(In the last 2 weeks)*** |  | *Jump to P8* | *Jump to P8* |
| - 1. Was the ulcer painful? |  |  |  |
| - 1. Did you receive treatment for the ulcer? |  | *Jump to P8* | *Jump to P8* |
| - - 1. Specify treatment:   ____________________________________________________________________________________ | | | |
| - - 1. Specify treatment date: __ __ __ __ / __ __ / __ __   *Y Y Y Y M M D D* | | | |
| 1. Has there been any ulcer in the perianal region?   ***(In the last 2 weeks)*** |  | *Jump to P9* | *Jump to P9* |
| - 1. Was the ulcer painful? |  |  |  |
| - 1. Did you receive treatment for the ulcer? |  | *Jump to P9* | *Jump to P9* |
| - - 1. Specify treatment:   ___________________________________________________________________________________ | | | |
| - - 1. Specify treatment date: __ __ __ __ / __ __ / __ __   *Y Y Y Y M M D D* | | | |
| 1. Has there been any ulcer in the oral region?   ***(In the last 3 months except the last 2 weeks)*** |  | *Jump to P10* | *Jump to P10* |
| - 1. Was the ulcer painful? |  |  |  |
| - 1. Did you receive treatment for the ulcer? |  | *Jump to P10* | *Jump to P10* |
| - - 1. Specify treatment:   _____________________________________________________________________________________ | | | |
| - - 1. Specify treatment date: __ __ __ __ / __ __ / __ __   *Y Y Y Y M M D D* | | | |

|  | **Yes** | | **No** | | **Doesn’t know** | |  |
| --- | --- | --- | --- | --- | --- | --- | --- |
| 1. Has there been any ulcer in the genital region?   ***(In the last 3 months except the last 2 weeks)*** |  | | *Jump to P11* | | *Jump to P11* | |  |
| - 1. Was the ulcer painful? |  | |  | |  | |  |
| - 1. Did you receive treatment for the ulcer? |  | | *Jump to P11* | | *Jump to P11* | |  |
| - - 1. Specify treatment:   ____________________________________________________________________________________ | | | | | | |  |
| - - 1. Specify treatment date. __ __ __ __ / __ __ / __ __   *Y Y Y Y M M D D* | | | | | | |  |
| 1. Has there been any ulcer in the perianal region?   ***(In the last 3 months except the last 2 weeks)*** | |  | | *Jump to P12* | | *Jump to P12* | |
| - 1. Was the ulcer painful? | |  | |  | |  | |
| - 1. Did you receive treatment for the ulcer? | |  | | *Jump to P12* | | *Jump to P12* | |
| - - 1. Specify treatment:   _____________________________________________________________________________________ | | | | | | | |
| - - 1. Specify treatment date. __ __ __ __ / __ __ / __ __   *Y Y Y Y M M D D* | | | | | | | |

1. **Secondary Syphilis symptoms**

| **In the last 3 months** | **Yes** | **No** | **Doesn’t know** |
| --- | --- | --- | --- |
| 1. Have you had fever? |  | *Jump to P13* | *Jump to P13* |
| - 1. How many days has it lasted?:_____________________, *(Integer values only)* | | | |
| 1. Have you had cutaneous eruption (rash) in palms and soles? |  | *Jump to P14* | *Jump to P14* |
| - 1. How many days has it lasted?:_____________________, *(Integer values only)* | | | |
| 1. Have you had cutaneous eruption (Rash) in trunk and back? |  | *Jump to P15* | *Jump to P15* |
| - 1. How many days has it lasted?:_____________________, *(Integer values only)* | | | |
| 1. Have you had loss of hair? |  | *Jump to P16* | *Jump to P16* |
| - 1. How many days has it lasted?:_____________________, *(Integer values only)* | | | |
| 1. Have you had any other symptom (potentially related to Syphilis)? |  | *Jump to P17* | *Jump to P17* |

| - 1. Any other symptom (potentially related to Syphilis   *Make a list of symptoms and complete the number of days each one last. | | |
| --- | --- | --- |
| N° | Symptoms | How many days has it lasted?  *(Integer values only)* |
| 1 |  |  |
| 2 |  |  |
| 3 |  |  |
| 4 |  |  |

1. **Nerosyphilis symptoms**

|  | **Yes** | **No** | **Doesn’t know** |
| --- | --- | --- | --- |
| 1. Have you have recent or new visual disturbances? |  | *Jump to P18* | *Jump to P18* |
| - 1. Complete duration of symptom in days | *(Numeric values only)* | | |
| 1. Have you have recent or new ocular pain? |  | *Jump to P19* | *Jump to P19* |
| - 1. Complete duration of symptom in days | *(Numeric values only)* | | |
| 1. Have you have recent or new loss of hearing? |  | *Jump to P20* | *Jump to P20* |
| - 1. Complete duration of symptom in days | *(Numeric values only)* | | |
| 1. Have you have recent or new vertigo? |  | *Jump to P21* | *Jump to P21* |
| - 1. Complete duration of symptom in days | *(Numeric values only)* | | |
| 1. Have you have recent or new headache? |  | *Jump to P22* | *Jump to P22* |
| - 1. Complete duration of symptom in days | *(Numeric values only)* | | |
| - 1. Headache intensity | *Ο 1 Ο 2 Ο 3 Ο 4 Ο 5*  *Ο 6 Ο 7 Ο 8 Ο 9 Ο 10* | | |
| - 1. Headache location | *Ο Global Ο Frontal Ο Occipital*  *Ο Temporal Ο Other* | | |
| 1. Have you have recent or new confusion episodes? |  | *Jump to P23* | *Jump to P23* |
| - 1. Complete duration of symptom in days | *(Numeric values only)* | | |
| 1. Have you have persistent and new or recent loss of strength or sensation in any body part? |  | *Jump to P24* | *Jump to P24* |
| - 1. Complete duration of symptom in days | *(Numeric values only)* | | |

1. **Other Sexually transmitted infections symptoms (In the last 3 months)**

| **In the last 3 months** | **Yes** | **No** | **Doesn’t know** | **No apply** |
| --- | --- | --- | --- | --- |
| 1. Have you had discharge throw your penis? *(men only)* |  |  |  |  |
| 1. Have you had vaginal discharge?   *(women only)* |  |  |  |  |
| 1. Have you had low abdominal pain?   (women only) |  |  |  |  |
| 1. Have you had dyspareunia?   (women only) |  |  |  |  |
| 1. Have you had dysuria? |  |  |  |  |
| 1. Have you had anal discharge? |  |  |  |  |
| 1. Have you had anal burning? |  |  |  |  |
| 1. Have you had rectal bleeding? |  | *Jump to P32* | *Jump to P32* | *Jump to P32* |
| - 1. Frequency | Ο Less than once a week  Ο Once a week  Ο 2 – 3 times a week  Ο 4 – 6 times a week  Ο Daily | | | |
| - 1. Describe blood volume | Ο Only drops or stains  Ο Less than 50 cc (3 or less soup spoons)  Ο 50-250 cc (from 3 soup spoons to one cup)  Ο More than 250 cc (more than a cup) | | | |
| 1. Have you had anal itching? |  |  |  |  |
| 1. Have you had swollen lymph nodes? |  |  |  |  |
| 1. Have you had any other symptom? |  | *Jump to P35* | *Jump to P35* | *Jump to P35* |
| - 1. Describe other symptoms | __________________________________________________  __________________________________________________  __________________________________________________ | | | |

1. **Physical examination**

|  | **Yes** | | **No** | **No examined** |
| --- | --- | --- | --- | --- |
| 1. Is there fever? |  | |  |  |
| 1. Is there palms/soles rash? |  | |  |  |
| 1. Is there trunk/body rash? |  | |  |  |
| 1. Is there lymphoadenomegaly? |  | | *Saltar a P40* | *Saltar a P40* |
| - 1. Describe location, size, consistence and pain | _________________________________________________________  _________________________________________________________  _________________________________________________________  _________________________________________________________ | | | |
| - 1. Is there cervical lymphadenomegaly? |  | | *Jump to P39* | *Jump to P39* |
| - - 1. Add oropharynx examination | _________________________________________________________  _________________________________________________________  _________________________________________________________  _________________________________________________________ | | | |
| - - 1. Add ear examination | _________________________________________________________  _________________________________________________________  _________________________________________________________  _________________________________________________________ | | | |
| 1. Is there alopecia? |  | |  |  |
| 1. Are there mucous patches? | |  |  |  |
| 1. Are there oral ulcers? | |  | *Jump to P42* | *Jump to P42* |
| - 1. Is there pain in oral ulcer(s)? | |  |  |  |
| 1. Are there non-oral/genital ulcers? | |  | *Jump to P43* | *Jump to P43* |
| - 1. Is there pain in non-oral/genital ulcer(s)? | |  |  |  |
| - 1. Add number and location of non-oral/genital ulcer | _________________________________________________________  _________________________________________________________  _________________________________________________________  _________________________________________________________ | | | |

|  | **Yes** | **No** | **No examined** |
| --- | --- | --- | --- |
| 1. Is there any other clinical finding? |  | *Jump to P44* | *Jump to P44* |
| - 1. Describe other clinical findings | _________________________________________________________  _________________________________________________________  _________________________________________________________  _________________________________________________________ | | |

1. **Anogenital examination**

|  | **Yes** | **No** | **No examined** |
| --- | --- | --- | --- |
| 1. Is there any penis or vulva ulcer? |  |  |  |
| 1. Is there any perineal ulcer? |  |  |  |
| 1. Is there any perianal ulcer? |  |  |  |
| 1. Is there any anal ulcer? (watched using anoscopes) |  |  |  |
| ***If at least one ulcer on penis, vulva, anus or perineal/perianal region answer question P47.1 and P47.2, otherwise jump to question P48.*** | | | |
| - 1. Complete how many ulcers were found on penis, vulva, anus or perineal/perianal region   _______________ (Numeric values only) | | | |
| - 1. Complete if there was pain on ulcers | | | |
| Ο Yes, in all of them Ο Yes, in some of them Ο None Ο No examined | | | |

1. **Current syphilis serology results, diagnostic and treatment**

| 1. RPR | | | | Ο Reactive Ο No reactive  ***(If “No reactive” Jump to P 50)*** | | | | | |
| --- | --- | --- | --- | --- | --- | --- | --- | --- | --- |
| 1. Complete current RPR result (dilutions) | | | | | | | |  |  |
| Ο 1:1 | Ο 1:2 | Ο 1:4 | Ο 1:8 | | | Ο 1:16 | Ο 1:32 | | |
| Ο 1:64 | Ο 1:128 | Ο 1:256 | Ο 1:512 | | | Ο 1:1024 |  | | |
| 1. Diagnosis   Ο Primary Syphilis  Ο Secondary Syphilis  Ο Early latent Syphilis  Ο Late/Of unknown duration latent syphilis  Ο Tertiary Syphilis (Suspected)**, explain reason in P50.1**  Ο Neurosyphilis (Suspected)**, explain reason in P50.1** | | | | | | | | | |
| - 1. Justify diagnosis of suspected tertiary Syphilis or Neurosyphilis   ____________________________________________________________________________________________________________________________________________________________________  __________________________________________________________________________________ | | | | | | | | | |
| 1. Which syphilis treatment regimen was prescribe today? | | | | | | | | | |
| Ο Penicillin G Benzathine 1 dose**, jump to P 51.2**  Ο Penicillin G Benzathine 3 doses**, jump to P 51.2**  Ο Doxycycline 100mg 2 weeks**, jump to P 51.5**  Ο Doxycycline 100mg 4 weeks**, jump to P 51.5**  Ο Another treatment**, complete another treatment in P 51.1** | | | | | | | | | |
| - 1. Which other treatment? *(complete medication name, dose and reason)*   _____________________________________________________________________________________________________________________________________________________________  __________________________________________________________________________________  ***End clinical examination providing sign and stamp from evaluator.*** | | | | | | | | | |
| Schedule dates for Penicillin injection and Doxycycline pills delivery | | | | | | | | | |
| - 1. First dose, ***(If it was prescribed Penicillin G Benzathine 1 or 3 doses)*** | | | | | Date: __ __ __ __ / __ __ / __ __  *Y Y Y Y M M D D* | | | | |
| - 1. Second dose, ***(If it was prescribed Penicillin G Benzathine 3 doses)*** | | | | | Date: __ __ __ __ / __ __ / __ __  *Y Y Y Y M M D D* | | | | |
| - 1. Third dose, ***(If it was prescribed Penicillin G Benzathine 3 doses)*** | | | | | Date: __ __ __ __ / __ __ / __ __  *Y Y Y Y M M D D* | | | | |
| - 1. Date of Doxycycline pills delivery ***(If it was prescribed Doxycycline 100mg 2 weeks or 4 weeks)*** | | | | | Date: __ __ __ __ / __ __ / __ __  *Y Y Y Y M M D D* | | | | |

_____________________________

**Sign and stamp from evaluator**

**CLINICAL FORM – FOLLOW UP, version 2**

1. **General information**

| Evaluator initials: ___________  (Write initials of first name and 2 last names) | Paste ID label here |
| --- | --- |
| Visit date : __ __ __ __ / __ __ / __ __  *Y Y Y Y M M D D* | |

1. **Morbidities**

| 1. Does the participant have any acute or chronic illness? | Ο Yes Ο No  ***(If “No” jump to P 2)*** |
| --- | --- |
| - 1. Write the illnesses the participant has: |  |
| ________________________________________________________________________________________  ________________________________________________________________________________________  ________________________________________________________________________________________ | |

1. **HIV infection history**

| 1. Is the participant HIV positive? | Ο Yes Ο No  ***(If “No” jump to P 3)*** |
| --- | --- |
| - 1. Is the participant currently on HIV treatment?   *(Take information from medical record. If there is no information on medical record, ask the participant)* | Ο Yes Ο No  ***(If “No” jump to P 3)*** |
| - - 1. Current antirretroviral regimen:   *(Take information from medical record. If there is no information on medical record, ask the participant)*   - ATRIPLA: Tenofovir 300 mg (TDF)/Emtricitabina 200 mg (FTC)/Efavirenz 600 mg (EFV)**, jump to P 3** - Tenofovir 300 mg (TDF)+Lamivudina 150 mg (3TC) +Efavirenz 600 mg (EFV)**, jump to P 3** - TRUVADA +EFAVIRENZ: Tenofovir 300 mg (TDF)/Emtricitabina 200 mg (FTC) +Efavirenz 600 mg (EFV)**, jump to P 3** - Another**, complete regimen in question P 2.1.1.1** - No information on medical record/Participant doesn’t remember**, jump to P 3** | |
| - - - 1. Specify other antiretroviral regimen: ________________________________________   ________________________________________________________________________________________________________________________________________________________________  *(Complete drug´s names and doses from clinical record. If there is no information on clinical record, ask the participant)* | |

1. **Syphilis history**

| 1. ¿Did the participant have a new diagnosis of Syphilis (reinfection) in the last study visit, except the first (basal) visit? | Ο Yes Ο No  ***(If “No” jump to P4)*** |
| --- | --- |
| - 1. ¿Which Syphilis treatment regimen was prescribe in the last study visit (regardless of compliance)? *(Take information from medical record. If there is no information on medical record, ask the participant)* | |
| Ο Penicillin G Benzathine 1 dose, **jump to P3.1.2 (Date of last treatment)**  Ο Penicillin G Benzathine 3 doses, **jump to P3.1.2 (Date of last treatment)**  Ο Doxycyclina 100mg 2 weeks, **jump to P3.1.2 (Date of last treatment)**  Ο Doxycyclina 100mg 4 weeks, **jump to P3.1.2 (Date of last treatment)**  Ο Otro Tratamiento, **complete another treatment in P 3.1.1**  Ο Do not remember /No information on clinical record, **jump to P3.1.2 (Date of last treatment)**  Ο Never treated, **jump to P 4** | |
| - - 1. Which another treatment did the participant receive in the last study visit?   __________________________________________________________________  __________________________________________________________________  __________________________________________________________________  *(Complete drug´s names and doses from clinical record. If there is no information on clinical record, ask the participant)* | |
| - - 1. Last syphilis treatment date from the last study visit | Date: __ __ __ __ / __ __ / __ __  *Y Y Y Y M M D D* |
| - 1. ¿Did the participant complete the previously mentioned treatment?   *(If treatment was Penicillin G Benzathine 1 dose, “Yes” and “I did not take/applied any of the treatment” are the only valid responses)* | |
| Ο Yes, ***jump to P 3.4 if treatment was Penicillin G Benzathine 1 or 3 doses; otherwise jump to P 4.***  Ο No, I took/applied only part of the treatment,  ***continue to P 3.3 if treatment was Penicillin G Benzathine 3 doses; otherwise jump to P 4.***  Ο *I did not take/applied any of the treatment****, jump to P 4*** | |
| - 1. ¿How many Penicillin doses did you receive? | Ο Only 1 dose  Ο Only 2 doses  Ο No apply *(If completed all doses of Penicillin treatment)* |
| - 1. ¿Did you receive your Penicillin single/first in your last study visit? | Ο Yes***, jump to P 3.6***  Ο No |
| - 1. ¿When did your received your Penicillin single/first dose? | Date: __ __ __ __ / __ __ / __ __  *Y Y Y Y M M D D* |
| - 1. ¿When did you received your Penicillin second dose?   *(Complete only if the participant completed treatment or received only 2 doses of Penicillin G Benzathine)* | Date: __ __ __ __ / __ __ / __ __  *Y Y Y Y M M D D* |
| - 1. ¿When did you received your Penicillin third dose?   *(Complete only if the participant complete treatment of Penicillin G Benzathine 3 doses)* | Date: __ __ __ __ / __ __ / __ __  *Y Y Y Y M M D D* |

| 1. ¿Did the participant had a new diagnosis of Syphilis outsite of the study site? | | Ο Yes Ο No Ο Don’t know  ***(If “No” or “Don’t know” jump to P 5)*** | | |
| --- | --- | --- | --- | --- |
| - 1. ¿Which was the last Syphilis treatment regimen the participant received outside the study site, after last study visit?   *(Take information from medical record. If there is no information on medical record, ask the participant)* | | | | |
| Ο Penicillin G Benzathine 1 dose, **jump to P4.1.2 (Date from last treatment)**  Ο Penicillin G Benzathine 3 doses, **jump to P4.1.2 (Date from last treatment)**  Ο Doxycyclina 100mg 2 weeks, **jump to P4.1.2 (Date from last treatment)**  Ο Doxycyclina 100mg 4 weeks, **jump to P4.1.2 (Date from last treatment)**  Ο Another Treatment, **complete another treatment in P 4.1.1**  Ο Don’t remember /No information in clinical record, **jump to P4.1.2 (Date from last treatment)**  Ο Never treated, jump to **P 5** | | | | |
| - - 1. Which another treatment did the participant receive?   __________________________________________________________________  __________________________________________________________________  __________________________________________________________________  *(Complete drug´s names and doses from clinical record. If there is no information on clinical record, ask the participant)* | | | |  |
| - - 1. Last syphilis treatment date | Date: __ __ __ __ / __ __ / __ __  *Y Y Y Y M M D D* | | |  |
| - - 1. Within 24 hours after taken/receiving syphilis treatment, in or out study visit, did the participant had new or more intensive symptoms of fever, malaise or cutaneous rash? | | | Ο Yes  Ο No  Ο No apply *(If didn’t take/receive any treatment in or out study visit)* | |
| 1. Is the participant allergic to Penicillin? | | Ο Yes Ο No | | |
| 1. Does the participant have any autoimmune disease such as lupus or rheumatoid arthritis? | | Ο Yes Ο No | | |

1. **Primary syphilis symptoms**

|  | **Yes** | **No** | **Doesn’t know** |
| --- | --- | --- | --- |
| 1. Has there been any ulcer in the oral region?   ***(In the last 2 weeks)*** |  | *Jump to P8* | *Jump to P8* |
| - 1. Was it a painful ulcer? |  |  |  |
| - 1. Did the ulcer receive treatment? |  | *Jump to P8* | *Jump to P8* |
| - - 1. Specify treatment:   ____________________________________________________________________________________ | | | |
| - - 1. Specify treatment date. __ __ __ __ / __ __ / __ __   *Y Y Y Y M M D D* | | | |

|  | **Yes** | **No** | **Doesn’t know** |
| --- | --- | --- | --- |
| 1. Has there been any ulcer in the genital region?   ***(In the last 2 weeks)*** |  | *Jump to P9* | *Jump to P9* |
| - 1. Was the ulcer painful? |  |  |  |
| - 1. Did you receive treatment for the ulcer? |  | *Jump to P9* | *Jump to P9* |
| - - 1. Specify treatment:   ____________________________________________________________________________________ | | | |
| - - 1. Specify treatment date: __ __ __ __ / __ __ / __ __   *Y Y Y Y M M D D* | | | |
| 1. Have you had an ulcer in the perianal region?   ***(In the last 2 weeks)*** |  | *Jump to P10* | *Jump to P10* |
| - 1. Was the ulcer painful? |  |  |  |
| - 1. Did you receive treatment for the ulcer? |  | *Jump to P9* | *Jump to P10* |
| - - 1. Specify treatment:   ___________________________________________________________________________________ | | | |
| - - 1. Specify treatment date: __ __ __ __ / __ __ / __ __   *Y Y Y Y M M D D* | | | |
| 1. Has there been any ulcer in the oral region?   ***(In the last 3 months except the last 2 weeks)*** |  | *Jump to P11* | *Jump to P11* |
| - 1. Was the ulcer painful? |  |  |  |
| - 1. Did you receive treatment for the ulcer? |  | *Jump to P11* | *Jump to P11* |
| - - 1. Specify treatment:     2. ___________________________________________________________________________________ | | | |
| - - 1. Specify treatment date: __ __ __ __ / __ __ / __ __   *Y Y Y Y M M D D* | | | |
| 1. Has there been any ulcer in the genital region?   ***(In the last 3 months except the last 2 weeks)*** |  | *Jump to P12* | *Jump to P12* |
| - 1. Was the ulcer painful? |  |  |  |
| - 1. Did you receive treatment for the ulcer? |  | *Jump to P12* | *Jump to P12* |
| - - 1. Specify treatment:   ____________________________________________________________________________________ | | | |
| - - 1. Specify treatment date. __ __ __ __ / __ __ / __ __   *Y Y Y Y M M D D* | | | |

|  | **Yes** | **No** | **Doesn’t know** |  |
| --- | --- | --- | --- | --- |
| 1. Has there been any ulcer in the perianal region?   ***(In the last 3 months except the last 2 weeks)*** |  | *Jump to P13* | *Jump to P13* | |
| - 1. Was the ulcer painful? |  |  |  | |
| - 1. Did you receive treatment for the ulcer? |  | *Jump to P13* | *Jump to P13* | |
| - - 1. Specify treatment:   _____________________________________________________________________________________ | | | | |
| - - 1. Specify treatment date. __ __ __ __ / __ __ / __ __   *Y Y Y Y M M D D* | | | | |

1. **Secondary Syphilis symptoms**

| **In the last 3 months** | | **Yes** | **No** | | **Doesn’t know** |
| --- | --- | --- | --- | --- | --- |
| 1. Have you had fever? | |  | *Jump to P14* | | *Jump to P14* |
| - 1. How many days has it lasted?:_____________________, *(Integer values only)* | | | | | |
| 1. Have you had cutaneous eruption (rash) in palms and soles? | |  | *Jump to P15* | | *Jump to P15* |
| - 1. How many days has it lasted?:_____________________, *(Integer values only)* | | | | | |
| 1. Have you had cutaneous eruption (Rash) in trunk and back? | |  | *Jump to P16* | | *Jump to P16* |
| - 1. How many days has it lasted?:_____________________, *(Integer values only)* | | | | | |
| 1. Have you had loss of hair? | |  | *Jump to P17* | | *Jump to P17* |
| - 1. How many days has it lasted?:_____________________, *(Integer values only)* | | | | | |
| 1. Have you had any other symptom (potentially related to Syphilis)? | |  | *Jump to P18* | | *Jump to P18* |
| - 1. Any other symptom (potentially related to Syphilis   *Make a list of symptoms and complete the number of days each one last. | | | | | |
| N° | Symptoms | | | How many days has it last?  (Interger values only) | |
| 1 |  | | |  | |
| 2 |  | | |  | |
| 3 |  | | |  | |
| 4 |  | | |  | |

1. **Nerosyphilis symptoms**

|  | **Yes** | **No** | **Doesn’t know** |
| --- | --- | --- | --- |
| 1. Have you have recent or new visual disturbances? |  | *Jump to P19* | *Jump to P19* |
| - 1. Complete duration of symptom in days | *(Numeric values only)* | | |
| 1. Have you have recent or new ocular pain? |  | *Jump to P20* | *Jump to P20* |
| - 1. Complete duration of symptom in days | *(Numeric values only)* | | |
| 1. Have you have recent or new loss of hearing? |  | *Jump to P21* | *Jump to P21* |
| - 1. Complete duration of symptom in days | *(Numeric values only)* | | |
| 1. Have you have recent or new vertigo? |  | *Jump to P22* | *Jump to P22* |
| - 1. Complete duration of symptom in days | *(Numeric values only)* | | |
| 1. Have you have recent or new headache? |  | *Jump to P23* | *Jump to P23* |
| - 1. Complete duration of symptom in days | *(Numeric values only)* | | |
| - 1. Headache intensity | *Ο 1 Ο 2 Ο 3 Ο 4 Ο 5*  *Ο 6 Ο 7 Ο 8 Ο 9 Ο 10* | | |
| - 1. Headache location | *Ο Global Ο Frontal Ο Occipital*  *Ο Temporal Ο Other* | | |
| 1. Have you have recent or new confusion episodes? |  | *Jump to P24* | *Jump to P24* |
| - 1. Complete duration of symptom in days | *(Numeric values only)* | | |
| 1. Have you have persistent and new or recent loss of strength or sensation in any body part? |  | *Jump to P25* | *Jump to P25* |
| - 1. Complete duration of symptom in days | *(Numeric values only)* | | |

1. **Other Sexually transmitted infections symptoms (In the last 3 months)**

| **In the last 3 months** | **Yes** | **No** | **Doesn’t know** | **No apply** |
| --- | --- | --- | --- | --- |
| 1. Have you had discharge throw your penis? *(men only)* |  |  |  |  |
| 1. Have you had vaginal discharge?   *(women only)* |  |  |  |  |
| 1. Have you had low abdominal pain?   (women only) |  |  |  |  |
| 1. Have you had dyspareunia?   (women only) |  |  |  |  |
| 1. Have you had dysuria? |  |  |  |  |
| 1. Have you had anal discharge? |  |  |  |  |
| 1. Have you had anal burning? |  |  |  |  |
| 1. Have you had rectal bleeding? |  | *Jump to P33* | *Jump to P33* | *Jump to P33* |
| - 1. Frequency | Ο Less than once a week  Ο Once a week  Ο 2 – 3 times a week  Ο 4 – 6 times a week  Ο Daily | | | |
| - 1. Describe blood volume | Ο Only drops or stains  Ο Less than 50 cc (3 or less soup spoons)  Ο 50-250 cc (from 3 soup spoons to one cup)  Ο More than 250 cc (more than a cup) | | | |
| 1. Have you had anal itching? |  |  |  |  |
| 1. Have you had swollen lymph nodes? |  |  |  |  |
| 1. Have you had any other symptom? |  | *Jump to P36* | *Jump to P36* | *Jump to P36* |
| - 1. Describe other symptoms | ____________________________________________________________________________________________________________________________________ | | | |

1. **Physical examination**

|  | **Yes** |  | **Yes** |
| --- | --- | --- | --- |
| 1. Is there fever? |  |  |  |
| 1. Is there palm/sole rash? |  |  |  |
| 1. Is there trunk/body rash? |  |  |  |
| 1. Is there lymphadenomegaly? |  | Jump to P40 | Jump to P40 |
| - 1. Describe location, size and consistence | _________________________________________________________  _________________________________________________________  _________________________________________________________  _________________________________________________________ | | |

|  | **Yes** | **No** | **No examined** |
| --- | --- | --- | --- |
| - 1. Is there cervical lymphadenomegaly? |  | *Jump to P40* | *Jump to P40* |
| - - 1. Add oropharynx examination | _________________________________________________________  _________________________________________________________  _________________________________________________________ | | |
| - - 1. Add ear examination | _________________________________________________________  _________________________________________________________  _________________________________________________________ | | |
| 1. Is there alopecia? |  |  |  |
| 1. Are there mucous patches? |  |  |  |
| 1. Are there oral ulcers? |  | *Jump to P43* | *Jump to P43* |
| - 1. Is there pain in oral ulcer(s)? |  |  |  |
| 1. Are there non-oral/genital ulcers? |  | *Jump to P44* | *Jump to P44* |
| - 1. Is there pain in non-oral/genital ulcer(s)? |  |  |  |
| - 1. Add number and location of non-oral/genital ulcer | _________________________________________________________  _________________________________________________________  _________________________________________________________ | | |
| 1. Are there any other clinical findings? |  | *Jump to P45* | *Jump to P45* |
| - 1. Describe other clinical findings | _________________________________________________________  _________________________________________________________  _________________________________________________________ | | |

1. **Anogenital examination**

|  | **Yes** | **No** | **No examined** |
| --- | --- | --- | --- |
| 1. Is there any penis or vulva ulcer? |  |  |  |
| 1. Is there any perineal ulcer? |  |  |  |
| 1. Is there any perianal ulcer? |  |  |  |
| 1. Is there any anal ulcer? (watch using anoscopes) |  |  |  |
| ***If at least one ulcer on penis, vulva, anus or perineal/perianal region answer question P47.1 and P47.2, otherwise jump to question P48.*** | | | |
| - 1. Complete how many ulcers were found on penis, vulva, anus or perineal/perianal region   _______________ (Numeric values only) | | | |
| - 1. Complete if there was pain in ulcer(s) | | | |
| Ο Yes, in all of them Ο Yes, in some of them Ο None Ο No examined | | | |

1. **Current syphilis serology results, diagnostic and treatment**

| 1. RPR | | | | Ο Reactive Ο No reactive  ***(If “No reactive” Jump to P 51)*** | | | | | |
| --- | --- | --- | --- | --- | --- | --- | --- | --- | --- |
| 1. Complete current RPR result (dilutions) | | | | | | | |  |  |
| Ο 1:1 | Ο 1:2 | Ο 1:1 | Ο 1:2 | | | Ο 1:1 | Ο 1:2 | | |
| Ο 1:64 | Ο 1:128 | Ο 1:64 | Ο 1:128 | | | Ο 1:64 | Ο 1:128 | | |
| 1. Diagnosis   (Do not complete with diagnosis from last study visits)  Ο Primary Syphilis  Ο Secondary Syphilis  Ο Early latent Syphilis  Ο Late/Of unknown duration latent syphilis  Ο Tertiary Syphilis (Suspected)**, explain reason in P51.1**  Ο Neurosyphilis (Suspected) **, explain reason in P51.1**  Ο None | | | | | | | | | |
| - 1. Justify diagnosis of suspected tertiary Syphilis or Neurosyphilis   ____________________________________________________________________________________________________________________________________________________________________  __________________________________________________________________________________ | | | | | | | | | |
| 1. Which syphilis treatment regimen was prescribe today? | | | | | | | | | |
| Ο Penicillin G Benzathine 1 dose**, jump to P 52.2**  Ο Penicillin G Benzathine 3 doses**, jump to P 52.2**  Ο Doxycycline 100mg 2 weeks**, jump to P 52.5**  Ο Doxycycline 100mg 4 weeks**, jump to P 52.5**  Ο Another treatment**, complete another treatment in P 52.1** | | | | | | | | | |
| - 1. Which other treatment? *(complete medication name, dose and reason)*   _____________________________________________________________________________________________________________________________________________________________  __________________________________________________________________________________  ***End clinical examination providing sign and stamp from evaluator.*** | | | | | | | | | |
| Schedule dates for Penicillin injection and Doxycycline pills delivery | | | | | | | | | |
| - 1. First dose, ***(If it was prescribed Penicillin G Benzathine 1 or 3 doses)*** | | | | | Date: __ __ __ __ / __ __ / __ __  *Y Y Y Y M M D D* | | | | |
| - 1. Second dose, ***(If it was prescribed Penicillin G Benzathine 3 doses)*** | | | | | Date: __ __ __ __ / __ __ / __ __  *Y Y Y Y M M D D* | | | | |
| - 1. Third dose, ***(If it was prescribed Penicillin G Benzathine 3 doses)*** | | | | | Date: __ __ __ __ / __ __ / __ __  *Y Y Y Y M M D D* | | | | |
| - 1. Date of Doxycycline pills delivery ***(If it was prescribed Doxycycline 100mg 2 weeks or 4 weeks)*** | | | | | Date: __ __ __ __ / __ __ / __ __  *Y Y Y Y M M D D* | | | | |

_____________________________

**Sign and stamp from evaluator**

**Risky behavior questionnaire, version 2**

| Interviewer initials: ___________  *(Write initials of first name and 2 last names)* | Paste ID label here | |
| --- | --- | --- |
| - 1. In the last 3 months, with how many women did you have sex? | | ___________  (Numeric values only) |
| - 1. In the last 3 months, with how many men did you have sex? | | ___________  (Numeric values only) |
| - 1. In the last 3 months, with how many transgender persons did you have sex? | | ___________  (Numeric values only) |
| - 1. In the last 3 months, how often have you used condoms when having anal or vaginal sex?   Ο Never  Ο Less than half the time  Ο Half the time  Ο More than half the time  Ο Always  Ο I didn’t have anal or vaginal sex in the last 3 months | | |
| - 1. In the last 3 months, which kind of condomless sex did you have?   (Check all that apply)   - Anal insertive (You insert your penis in other men/women’s anus) - Anal receptive (Penis was insert in your anus) - Vaginal insertive (You insert your penis in woman/trans women’s vagina) - Vaginal receptive (Penis was insert in your vagina) | | |
| - 1. ¿Have you ever taken pre-exposure prophylaxis (PrEP)? | | Ο In the past Ο Currently Ο Never |

_____________________________

**Coordinator sign and stamp**

**Demographic questionnaire – basal, version 3**

| Interviewer initials: ___________  (Write initials of first name and 2 last names) | Paste ID label here |
| --- | --- |
| Visit date : __ __ __ __ / __ __ / __ __  *Y Y Y Y M M D D* | |
| Recruitment site:  Ο Barton Ο Tahuantinsuyo Ο Caja de agua Ο Epicentro Ο Pucallpa | |
| Enrollment arm:  Ο A: Participant with previous syphilis infection  Ο B: Participant without previous syphilis infection  Ο C: Participant with unknown syphilis infection history | |

1. **Sociodemographic characteristics**

| 1. Birth date: | | | __ __ __ __ / __ __ / __ __  *Y Y Y Y M M D D* | |
| --- | --- | --- | --- | --- |
| 1. Sex assigned at birth | | | Ο Male Ο Female | |
| 1. ¿Do you consider yourself a transgender/transsexual/travesti? | | | Ο Yes Ο No | |
| 1. ¿Which of the following terms do you think represent better your sexual identity?   Ο Heterosexual Ο Gay Ο lesbian Ο Bisexual Ο Other | | | | |
| 1. Educational attainment   Ο Incomplete primary school  Ο Complete primary school  Ο Incomplete secondary school  Ο Complete secondary school  Ο Incomplete higher technological education  Ο Complete higher technological education  Ο Incomplete university education  Ο Complete university education | | |  | |
| 1. District of residence | | | | |
| **In Lima** | | | | |
| Ο Ancón  Ο Ate  Ο Barranco  Ο Breña  Ο Callao  Ο Carabayllo  Ο Chaclacayo  Ο Chorrillos  Ο Cieneguilla  Ο Comas  Ο El Agustino  Ο Independencia | Ο Jesús María  Ο La Molina  Ο La Victoria  Ο Lima  Ο Lince  Ο Los Olivos  Ο Lurigancho-Chosica  Ο Lurín  Ο Magdalena Del Mar  Ο Miraflores  Ο Pachacamac  Ο Pucusana | Ο Pueblo Libre  Ο Puente Piedra  Ο Punta Hermosa  Ο Punta Negra  Ο Rímac  Ο San Bartolo  Ο San Borja  Ο San Isidro  Ο San Juan De Lurigancho  Ο San Juan De Miraflores  Ο San Luis  Ο San Martin De Porres | | Ο San Miguel  Ο Santa Anita  Ο Santa María Del Mar  Ο Santa Rosa  Ο Santiago De Surco  Ο Surquillo  Ο Villa El Salvador  Ο Villa María Del Triunfo  Ο Another***, Complete question P 6.1*** |
|  |  |  | |  |
| **In Ucayali: Coronel Portillo (CP), Atalaya, Padre Abad (PA), Purús** | | | | |
| Ο Purús-Purús | Ο Callería-CP (Pucallpa) | Ο Iparia-CP | | Ο Yarinacocha-CP |
| Ο Manantay-CP | Ο Campoverde-CP | Ο Masisea-CP | | Ο Nueva Requena-CP |
| Ο Seáhua-Atalaya | Ο Tahuanita-Atalaya | Ο Yurua-Atalaya | | Ο Raymondi-Atalaya |
|  |  |  | |  |
| Ο Irazola- PA | Ο Curimana-PA | Ο Neshuya- PA | | Ο Alexander Von Humbolt- PA |
| Ο Padre Abad-PA | Ο Another, ***Complete question P 6.1*** |  | |  |
| - 1. Complete "another" residence location:_____________________________________________________ | | | | |
| 1. ¿Do you have health insurance?   Ο SIS Ο EsSalud Ο Another Ο None | | | |  |

1. **HIV testing and diagnosis**

| 1. ¿When was your last HIV test?   Ο Less than 2 months ago  Ο From 2 to 6 months ago  Ο From more than 6 months to 12 months ago  Ο More than 12 months ago  Ο I’ve never been tested***. End questionnaire.*** | | |
| --- | --- | --- |
| - 1. ¿Which was your last HIV result? | Ο Positive Ο Negative Ο I don’t know  ***If “Negative” or “I don’t know”, end questionnaire.*** | |
| - - 1. Date of diagnosis | __ __ __ __ / __ __ / __ __  *Y Y Y Y M M D D* | |
| - 1. ¿When was your first CD4 count taken?   Ο Less than 2 months ago  Ο From 2 to 6 months ago  Ο From more than 6 months to 12 months ago  Ο More than 12 months ago  Ο I’ve never been tested***, jump to P 8.3*** | | |
| - - 1. Which was your initial CD4 count value?   Ο < 200 Ο 200-350 Ο 350-500 Ο >500 Ο I don’t know | | |
| - 1. ¿Are you currently taking anti-retroviral treatment? | | Ο Yes Ο No |
| - 1. ¿Is your viral load undetectable? | | Ο Yes Ο No Ο I don’t know |

_____________________________

**Coordinator sign and stamp**

**Demographic questionnaire – Follow up, version 2**

| Interviewer initials: ___________  (Write initials of first name and 2 last names) | Paste ID label here |
| --- | --- |
| Visit date: __ __ __ __ / __ __ / __ __  *Y Y Y Y M M D D* | |

**HIV history**

| 1. ¿Which is your HIV status? | Ο Positive Ο Negative Ο I don’t know  ***If “negative” or “I don’t know”, end questionnaire.*** |
| --- | --- |
| - 1. ¿Are you currently taken antirretroviral treatment? | Ο Yes Ο No  ***If “No”, end questionnaire*** |
| - - 1. In the last 3 months, have you ever forgot to take your HIV medication? | Ο Yes Ο No |
| - - 1. In the last 3 months, have you ever stop taking your HIV medication because you felt ill? | Ο Yes Ο No |
| - - 1. ¿Did you forgot to take your HIV medication in the last weekend? | Ο Yes Ο No |
| - - 1. In the last week, how many times did you not take a dose?   Ο Never Ο 1-2 Ο 3-5 Ο 6-10 Ο More than 10 | |
| - - 1. In the last 3 months, how many complete days did you not take your HIV medication? | ___________  (Numeric values only) |

_____________________________

**Coordinator sign and stamp**

**Questionnaire – 4 weeks, version 1**

| Interviewer initials: ___________  *(Write initials of first name and 2 last names)* | Paste ID label here |
| --- | --- |
| Date of interview : __ __ __ __ / __ __ / __ __  *Y Y Y Y M M D D* | |

1. **Morbidities**

| 1. ¿Does the participant currently have any acute or chronic disease?   (Including previously diagnosed HIV) | Ο Yes Ο No |
| --- | --- |
| - 1. Complete the diseases the participant has: _________________________________________________   _________________________________________________________________________________  _________________________________________________________________________________  _________________________________________________________________________________  _________________________________________________________________________________  _________________________________________________________________________________ | |

1. **Syphilis treatment from first visit**

| 1. ¿Which treatment were you assigned in your first study visit (regardless of start or compliance of treatment)?   Ο Benzathine Penicillin G 1 dose  Ο Benzathine Penicillin G 3 doses  Ο Doxycycline 100mg 2 weeks  Ο Doxycycline 100mg 4 weeks  Ο Other Treatment | |
| --- | --- |
| 1. ¿Did you complete the aforementioned syphilis treatment?   *(If treatment was Penicillin G Benzathine 1 dose, it is only valid to mark "Yes" or "I did not take treatment/I did not received any treatment")* | |
| Ο Yes,  Ο No, I took/I received only part of the treatment  Ο No I did not take/I did not received any treatment***, End the questionnaire.*** | |
| 1. Within 24 hours after taking/receiving treatment for syphilis, inside or outside the study, did you have new or more intense to previous symptoms of fever, malaise or rash on the body? | Ο Yes Ο No |

1. **Compliance of Penicillin G Benzathine treatment prescribe in first study visit**

| - The following questions should only be fill if the participant received at least one dose of penicillin G Benzathine 1 dose or 3 doses. - If the participant answered “Penicillin G Benzathine 3 doses” in question P2, and “No, I took/I received only part of the treatment” in question P3, question P5 must be answer, otherwise jump to question P6. | |
| --- | --- |
| 1. ¿How many doses of penicillin did you received? | Ο Only 1 dose  Ο Only 2 doses |
| 1. ¿Did you receive the only/first dose of Penicillin the day of your last visit? | Ο Si***, jump to P 7***  Ο No |
| - 1. ¿When did you receive your only/first dose of Penicillin? | Date: __ __ __ __ / __ __ / __ __  *Y Y Y Y M M D D* |
| 1. ¿When did you receive your second dose of Penicillin? *(Answer only if participant completed treatment or receive only 2 dose Penicillin G Benzathine)* | Date: __ __ __ __ / __ __ / __ __  *Y Y Y Y M M D D* |
| 1. ¿When did you receive your third dose of Penicillin? *(Answer only if participant complete treatment of Penicillin G Benzathine 3 doses)* | Date: __ __ __ __ / __ __ / __ __  *Y Y Y Y M M D D* |

_____________________________

**Coordinator signature and stamp**
